# The Integrated Genomic Architecture and Evolution of Dental Divergence in East African Cichlid Fishes (*Haplochromis chilotes* x *H. nyererei*)

**DOI:** 10.1534/g3.117.300083

**Published:** 2017-07-27

**Authors:** C. Darrin Hulsey, Gonzalo Machado-Schiaffino, Lara Keicher, Diego Ellis-Soto, Frederico Henning, Axel Meyer

**Affiliations:** * Department of Biology, University of Konstanz, 78457, Germany; † Department of Genetics, Federal University of Rio de Janeiro, 21941, Brazil

**Keywords:** adaptive radiation, key innovation, natural selection, QTL, trophic evolution

## Abstract

The independent evolution of the two toothed jaws of cichlid fishes is thought to have promoted their unparalleled ecological divergence and species richness. However, dental divergence in cichlids could exhibit substantial genetic covariance and this could dictate how traits like tooth numbers evolve in different African Lakes and on their two jaws. To test this hypothesis, we used a hybrid mapping cross of two trophically divergent Lake Victoria species (*Haplochromis chilotes* × *Haplochromis nyererei*) to examine genomic regions associated with cichlid tooth diversity. Surprisingly, a similar genomic region was found to be associated with oral jaw tooth numbers in cichlids from both Lake Malawi and Lake Victoria. Likewise, this same genomic location was associated with variation in pharyngeal jaw tooth numbers. Similar relationships between tooth numbers on the two jaws in both our Victoria hybrid population and across the phylogenetic diversity of Malawi cichlids additionally suggests that tooth numbers on the two jaws of haplochromine cichlids might generally coevolve owing to shared genetic underpinnings. Integrated, rather than independent, genomic architectures could be key to the incomparable evolutionary divergence and convergence in cichlid tooth numbers.

The subdivision of organisms into distinct developmental genetic modules or units greatly influences the evolution of biological diversity. Similar to processes such as speciation (Slowinski and Guyer 1993; Elmer *et al.* 2014) and gene duplication (Meyer and Schartl 1999; Taylor *et al.* 2001; Wagner 2008; Ohno 2013) that both increase the particulate nature of biological entities, segmentation of organisms into serially homologous units has been suggested to be a major facilitator of diversification (Wagner 1996). When particular plant leaves, arthropod limbs, or vertebrate teeth are able to evolve independently and subsequently become specialized with respect to other homologous organs, this can heavily influence a clade’s evolutionary success (Wagner and Altenberg 1996; Stock 2001; Edwards and Weinig 2011; Santana and Lofgren 2013; Miller *et al.* 2014). For instance, the capacity of the two jaws of cichlid fishes to evolve independently has been commonly invoked as a critical catalyst underlying both the unparalleled trophic divergence of these fishes and the phenomenal convergence that characterizes their adaptive radiations (Liem 1973; Hulsey *et al.* 2006). However, the degree to which particular parts of phenotypes can evolve independently and contribute to adaptation might be largely a result of their underlying genetic architectures (Lande 1984; Albertson and Kocher 2006; Brakefield 2006; Wilkens 2010; Albertson *et al.* 2014; Armbruster *et al.* 2014; Gross *et al.* 2016). Structures ranging from flower pistils to snake vertebrae to primate teeth have been suggested to evolve largely according to their genetic covariance (Schluter 1996; Hohenlohe and Arnold 2008; Hlusko 2016; Smith 2016). Likewise, genomic architecture may have constrained even the astounding phenotypic diversity of cichlid teeth. To better understand the factors structuring trophic evolution in one of the most species-rich vertebrate radiations on Earth, we examined the genomic substrate of tooth number divergence on both the oral and pharyngeal jaws of East African cichlids.

Most groups of fish have two toothed jaws (Schaeffer and Rosen 1961; Figure 1). They have oral jaws that are largely homologous to our jaws and are used primarily to capture prey, and they also have pharyngeal jaws, which are modified gill arches, that are used to macerate and process prey (Liem 1973). However, unlike any other group of fish, cichlids exhibit an incredible amount of diversity in the shape, size, and number of teeth on both of their jaws (Fryer and Iles 1972). Because several lines of evidence have supported the functional as well as the evolutionary independence of the two jaws (Liem 1973; Hulsey *et al.* 2006), cichlid oral and pharyngeal teeth might commonly change independently to promote the distinct trophic roles of their two jaws. Divergence in teeth could most often be the result of genes that are specifically involved in forming teeth on only one jaw. Yet at least 30% of the genes found to be involved in forming mouse teeth are also expressed in both tooth-bearing jaws of cichlids (Hulsey *et al.* 2016). This suggests that there is likely a substantial degree of developmental similarity, or integration at the level of gene expression, in teeth on these morphologically and evolutionarily distinct jaws. Also, despite the distinct evolutionary origin of the two sets of jaws, the evolution of tooth number on both the oral and pharyngeal jaws across a diversity of Malawi cichlids is highly and positively correlated (Fraser *et al.* 2009). Therefore, the genomic architecture dictating dental divergence on both cichlid jaws could be highly similar.

**Figure 1:**
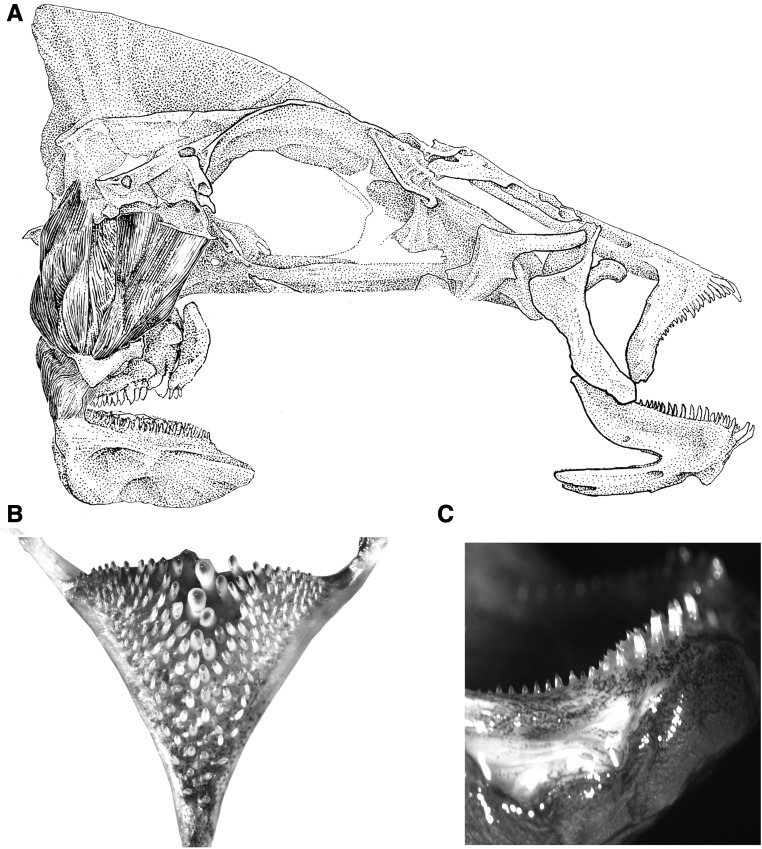
The toothed oral and pharyngeal jaws of cichlids (A). Dorsal view of the pharyngeal tooth plate of an F2 hybrid is displayed (B) and the large numbers of teeth that can be counted on the jaw are readily seen. The oral jaw dentition (C) of an F2 hybrid cichlid in lateral view.

Cichlid fishes are well known for the repeated independent evolution of similar features in different lineages (Fryer and Iles 1972; Rüber *et al.* 1999; Brawand *et al.* 2014). Their rampant convergence is also often used as an iconic example of how natural selection can deterministically shape diversity (Henning and Meyer 2014; Machado-Schiaffino *et al.* 2015). For instance, in environments such as Lakes Victoria and Malawi, phylogenetically distinct lineages of cichlids have colonized each lake independently from riverine environments and then, within very short timeframes, repeatedly evolved similar feeding specializations (Meyer *et al.* 1990; Meyer 1993; Genner *et al.* 2007; Hulsey *et al.* 2010). These convergent trophic phenotypes such as algae scraping, mollusk-crushing, and piscivory generally exhibit a suite of similarities in traits such as body shape and pigmentation, as well as the dentition on both their oral and pharyngeal jaws (Streelman *et al.* 2003; Henning *et al.* 2014; Machado-Schiaffino *et al.* 2015). As an example, piscivorous species have repeatedly evolved oral and pharyngeal jaws that display < 100 teeth, while herbivorous cichlids often possess several hundred teeth on both jaws (Fryer and Iles 1972; Fraser *et al.* 2009). Natural selection may be so strong that the repeated correspondence in tooth numbers on both jaws would depend very little on how similar the genomic underpinnings for teeth are, and would be wholly the result of adaptive processes. For instance, having a large number of teeth on the oral jaws might simply be necessary for scraping algae from the substrate, while for the same species having a large number of pharyngeal teeth could be ideal for breaking down algae for digestion (Fermon and Cibert 1998; Rupp and Hulsey 2014). However, convergent oral and pharyngeal tooth numbers in multiple lineages would also have been easier to evolve if similar loci controlled tooth number on both jaws.

The repeated emergence of convergent phenotypes among relatively closely related lineages could frequently result from parallel evolution that arises owing to changes in similar loci (Schluter *et al.* 2004; Shapiro *et al.* 2006; Stern and Orgogozo 2008; Rosenblum *et al.* 2010; Stern 2013; O’Brown *et al.* 2015). However, even among close relatives, the molecular basis of adaptation can differ markedly (Beall 2007; Ellis *et al.* 2015). Also, most documented examples of parallel evolution come from low-diversity systems with only a few species (Stern 2013), and therefore, parallelism could be rare in the extraordinarily species-rich East African Rift Lakes. Although Lake Malawi and Lake Victoria each contain several hundred species, all of these species have diverged extremely rapidly (∼2 MYA) from a common ancestor (Genner *et al.* 2007; Friedman *et al.* 2013). Interspecific gene flow among many of these recently diverged lineages also still occurs occasionally (Mims *et al.* 2010; Holzman and Hulsey 2017). These aspects of their evolutionary past have likely contributed to the considerable sharing of ancestral molecular polymorphisms observed even among cichlid lineages that are geographically separated (Loh *et al.* 2008; Brawand *et al.* 2014). However, whether or not these shared ancestral polymorphisms contribute to phenotypic divergence or even repeatedly evolved parallel adaptations is not known. But because our understanding of genomic architecture is rapidly increasing in African cichlid radiations (Brawand *et al.* 2014; Henning and Meyer 2014), we can now begin to compare whether similar genomic regions do underlie divergence in similar phenotypes between evolutionarily distinct clades of cichlids. For instance, several quantitative trait loci (QTL) for oral jaw tooth number were recently identified in a hybrid cross of Lake Malawi cichlids (Bloomquist *et al.* 2015), and these same loci might be playing a part in tooth divergence in many closely related cichlids. To test this idea, additional hybrid crosses of species with different dentitions could be used to determine whether the same genomic regions responsible for phenotypic divergence in tooth number are shared across distinct cichlid clades.

The genetic underpinnings of traits are often thought to influence or even constrain how they diversify (Lande 1984; Schluter 1996). However, there are very few examples where the genetic correlations among traits are known in similar taxa in which we understand the degree to which traits coevolve over phylogenetic scales (Brakefield 2006). Yet we are increasingly dissecting the genomic basis of traits such as flower pistil and stamen lengths, mammalian limb lengths, and the size of different hominid teeth, which have also been examined in a phylogenetically informed context (Hlusko 2016; Smith 2016). Combining genetic crosses with comparative analyses, should allow us to determine whether levels of genomic integration can predict the degree to which traits change in concert across their macro-evolutionary history. For instance, if the same loci are influencing traits such as tooth number across a phylogenetic diversity of cichlid species, it is possible to ask whether the phylogenetic correlations in tooth phenotypes mirror the observed correlations among hybrid crosses. Shared genomic architecture could be critical to explanations of the evolution of an adaptive phenotype as iconic as cichlid teeth.

Using a hybrid cross of two species of Lake Victoria haplochromine cichlid, we examined QTL associated with several dental traits. First, we asked whether there is any evidence for a shared genetic basis for tooth number in the oral jaws of Malawi cichlids and Victorian cichlids. Then, we screened our F2 mapping panel for QTL associated with several traits putatively related to tooth number on the pharyngeal jaw and examined the correlations among tooth traits. We also tested whether there is any evidence for colocalization of the genetic basis for tooth number divergence on both the oral and pharyngeal jaws. Finally, because we found a strong correlation between the number of teeth on both the oral and pharyngeal jaws in our hybrid population of Lake Victoria cichlids, we tested whether this correlation was statistically different from the correlation observed for the two jaws across a phylogenetic breadth of the closely related Lake Malawi cichlids.

## Materials and Methods

To examine the quantitative genetics of cichlid dentitions, we used a hybrid cross between two closely related Lake Victoria cichlids. A male *Haplochromis chilotes* and a female *H. nyererei* were crossed initially. When the female was identified as mouth-brooding, she was transferred to another 360 L tank. The resulting F1 generation was raised to sexual maturity (∼6–9 months). Subsequently, three F1 females were crossed with a single F1 male to generate F2 offspring. All the F2 hybrids were raised in partitions of 360 L aquaria. The sizes of the partitions were adjusted according to family size to ensure that all F2 were raised under similar densities and to minimize environmental effects on phenotypes. This cross was initially formed to examine the genetics of the hypertrophied lips of *Haplochromis chilotes* (Henning *et al.* 2017). Following completion of that study, it was observed that the two parental species differed in tooth number on both jaws. On the right dentary of the lower oral jaw, the parental *H. chilotes* individual possessed only three teeth, while the *H. nyererei* individual had 12 teeth in the same location. On their lower pharyngeal jaws, *H. chilotes* exhibited 112 teeth, while the *H. nyererei* had a total of 193 teeth. These differences in tooth numbers suggested this genetic cross could provide insight into the genomic basis of dental divergence.

Our analyses included 177 individuals genotyped previously in Henning *et al.* (2017). We augmented these previously genotyped individuals with 50 additional F2 from the same parental cross. To generate genotypes, genomic DNA was isolated from fin tissues of the 227 F2, four F1, and both parental individuals using the Zymo extraction kit (Zymo Research). The genotypes for the 50 newly phenotyped F2 individuals were generated solely for this study. Prior to genotyping, DNA integrity was evaluated with both agarose gel electrophoresis and a QUBIT v2.0 fluorometer (Life Technologies, Germany). Then, to generate RADseq libraries, we followed the methodology described in Peterson *et al.* (2012). For the libraries, 1 μg of Genomic DNA per sample was first double-digested using the restriction enzymes *Pst*I and *Msp*I (New England BioLabs) for 3 hr at 37 °C. P1 and P2 adapters were then ligated to the digested DNA using T4 ligase for 30 min at room temperature. Sequence fragment size selection was performed using Pippin Prep (Sage Science) with a range of 325–400 bp. Individually barcoded samples were then genotyped in pooled Illumina libraries. All genomic libraries were single-end sequenced (101 bp length) on the Illumina HiSeq 2000 platform.

The linkage map we used was originally constructed for the QTL mapping of several other traits and is described fully in Henning *et al.* (2017). In brief, marker genotypes were identified from Illumina raw reads with the STACKS pipeline (Catchen *et al.* 2013). The genotypic data were filtered according to previous guidelines for RAD linkage maps that permitted no more than 20% missing genotypes, as well as limited levels of marker segregation distortion (Henning *et al.* 2014). The JoinMap v.4 software (Van Ooijen 2006) was used to construct the linkage map. Quality control of this map was performed through inspection of graphical genotypes (Van Ooijen and Jansen 2013), recombination counts (Broman and Sen 2009a), and congruence between estimation methods, as well as published cichlid genetic and physical maps (Brawand *et al.* 2014; Franchini *et al.* 2014). The linkage map had a total inferred size of 1225.68 cM and was blanketed with 1122 ddRAD markers distributed across 22 linkage groups (Henning *et al.* 2017), which is in agreement with the haploid chromosome number in haplochromine cichlids (Thompson 1981). Additionally, there was substantial correspondence between the linkage map and the *P. nyererei* draft genome sequence (Brawand *et al.* 2014), allowing for high-quality anchoring of scaffolds to our linkage map (Supplemental Material, File S1). Our newly generated Illumina reads were incorporated into this high-quality linkage map to allow the robust estimation of dental QTL.

To control for the size of individuals, we measured the standard length (SL) of fish using dial calipers. Sex of individuals was also taken from Henning *et al.* (2017) where appropriate, and for newly genotyped individuals, determined from known differences in cichlid reproductive phenotypes (Carruth 2000). Then, the same researcher counted teeth on both oral and pharyngeal jaw elements. Although these species both had multiple rows of teeth in their oral jaws, the hybrids only had prominent teeth on the first tooth row. Therefore, for the oral jaw tooth phenotype, the number of teeth on only the first row of the bottom right dentary was counted under a dissecting microscope. To determine pharyngeal jaw phenotypes, we dissected the fifth ceratobranchial from the fish. These bony elements were then cleaned of all muscle and fascia and allowed to dry. Then, a size-standardized digital image of the dorsal surface of the jaws was taken and imported into the program ImageJ (Schneider *et al.* 2012). The area from a dorsal view encircling all of the teeth on the lower pharyngeal jaw was measured as tooth plate area. Additionally, because it allowed the strongest inference of homology among individuals, we measured the posterior-most tooth to the right of the pharyngeal jaw midline to determine pharyngeal tooth area. These two area measurements were square root transformed. Finally, we digitally counted the number of teeth on the lower pharyngeal jaw. For those traits that were correlated with SL, the correlation’s residuals were used in all QTL analyses. Correlations of tooth trait phenotypes in the F2 recombinant population were also determined to evaluate how genetically integrated the traits were in the hybrid cross.

Trait mapping was done in two steps. Linkage mapping was performed for each focal trait using the interval mapping algorithm implemented in R/qtl (Broman and Sen 2009b). Subsequently, QTL were corroborated with composite interval mapping in WinQTL Cartographer (Wang *et al.* 2012). The cichlid linkage groups were reported with reference to the closely related Nile Tilapia, *Oreochromis niloticus*, candidate intervals and chromosomes (Brawand *et al.* 2014; Conte *et al.* 2017). To test the significance of QTL, logarithm of odds (LOD) threshold peaks were calculated using 1000 permutations and a significance level of *P* < 0.05. The 1.5 LOD intervals that bounded identified QTL were also determined using r/qtl to assess whether QTL for different dental traits overlapped. For traits showing significant or highly suggestive QTL, we also blocked by sex to determine whether sex alone could explain genetic associations with trait values. The approximate lengths in bp that identified QTL regions encompassed were also calculated from the recently resequenced Tilapia genome (Conte *et al.* 2017). Additionally, several genes contained within these regions that are thought to play a part in vertebrate tooth development were highlighted (Hulsey *et al.* 2016).

The Pearson correlation coefficient of oral and pharyngeal jaw tooth numbers from the hybrid population, *r_q_*, was also statistically compared with the phylogenetically corrected Pearson correlation coefficient of independent contrasts, *r_p_*, estimated for oral and pharyngeal tooth numbers in Lake Malawi cichlids (Fraser *et al.* 2009). Because both correlation coefficients determine how much change in tooth number on one jaw explains change in tooth number on the other jaw, we determined whether these two correlations described similar relationships for changes in tooth numbers. To do this, we applied Fisher’s *z*-transformation and estimated the 95% confidence interval for the difference between the correlation coefficients, as described in Zou (2007). We implemented this test in the R package cocor (Diedenhofen and Musch 2015) to test for a significant difference between *r_q_* and *r_p_*.

### Data availability

The linkage map utilized is available in the supplementary materials of Henning *et al.* (2017). The reconstructed linkage groups, their genetic distance, and sequences used to reconstruct the linkage map are provided (File S1). All trait data are also available as are the genotype scores for each individual (File S2).

## Results

There was substantial morphological variability in the hybrid cross population. The F2 fish ranged from 55 to 118 mm in SL (File S2). Tooth numbers on the right side of the lower oral jaw in the first tooth row ranged from 1 to 24 teeth. The tooth plate area varied between 5.06 and 20.65 mm^2^, and pharyngeal tooth area was found to extend from 0.01 to 0.28 mm^2^ in the F2 hybrids. Lower pharyngeal jaw tooth numbers ranged from 64 to 230. Pharyngeal tooth plate area (*r* = 0.911, *P* < 0.001) and pharyngeal tooth area (*r* = 0.622, *P* < 0.001) were both strongly correlated with SL. However, there was no correlation between either pharyngeal tooth plate area (*P* = 0.130) or pharyngeal tooth area (*P* = 0.190) and the number of lower pharyngeal jaw teeth. Despite having 231 individual data points, neither oral (*r* = −0.002, *P* = 0.976) nor pharyngeal jaw (*r* = 0.016, *P* = 0.813) tooth numbers showed a correlation with SL. Raw counts of tooth numbers were therefore used in all further analyses.

**Figure 2:**
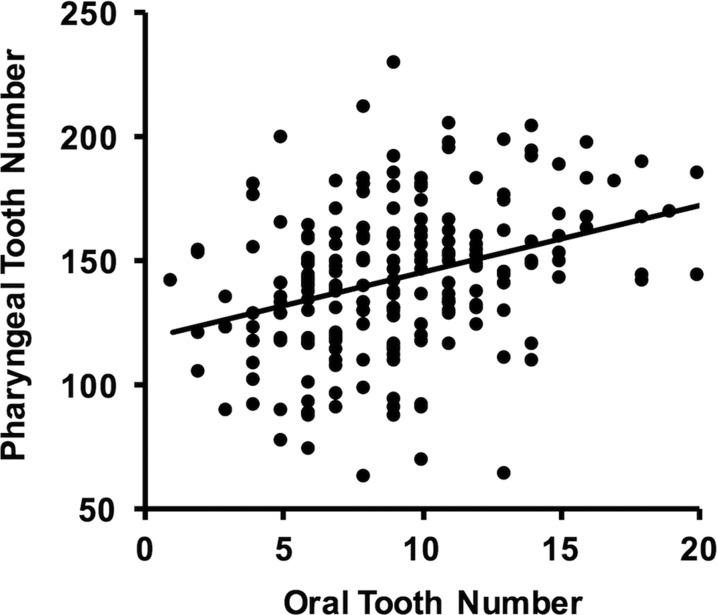
Correlation of tooth numbers in the oral and pharyngeal jaws of 227 F2 hybrids. The number of teeth in the first row on the right hand side of the lower tooth region of the oral jaw and all of the teeth on the dorsal surface of the lower pharyngeal jaw were counted in the F2 individuals. Tooth numbers on the two jaws were significantly correlated (*r_q_* = 0.35, *P* < 0.0001).

**Figure 3:**
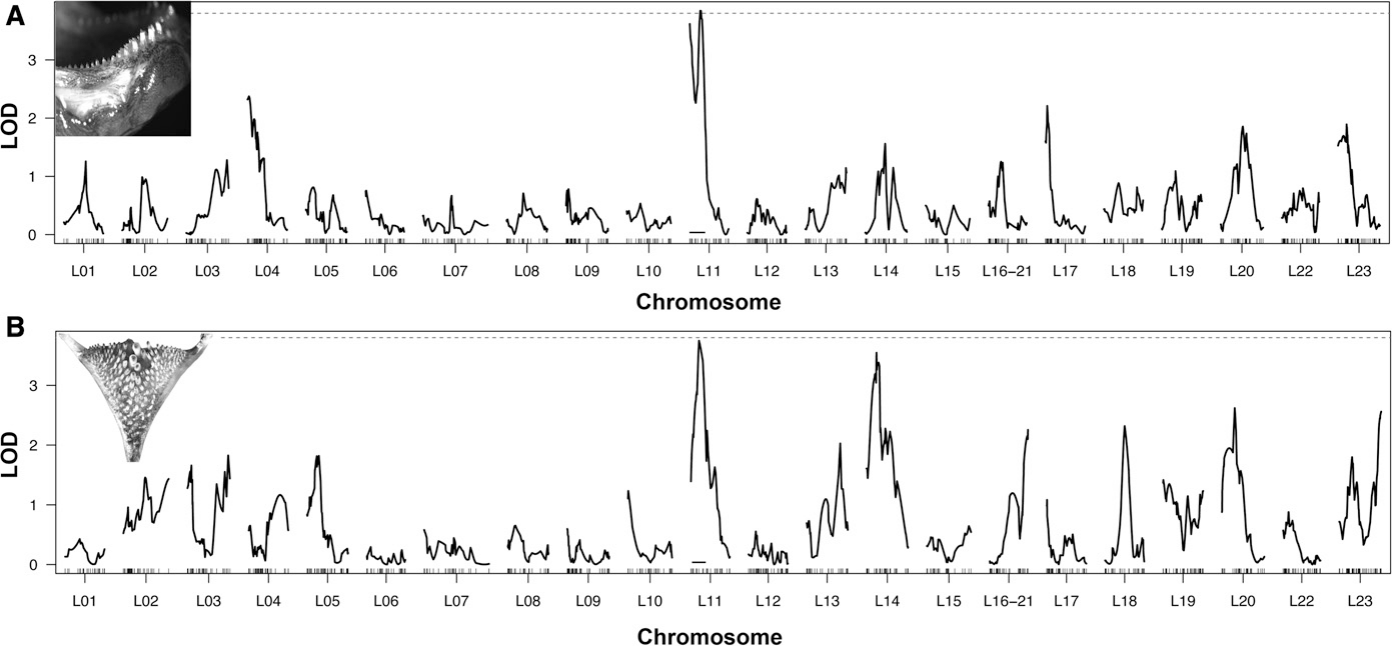
Tooth number QTL in the oral and the pharyngeal jaws. Linkage group numbers are given as the homologized regions of the Tilapia cichlid genome. The level of significance determined from permutations is shown with a dotted line. Both the oral (A) and pharyngeal jaws (B) have a large-effect QTL on chromosome 11. The 1.5 LOD intervals are highlighted with a black line. These QTL are colocalized to the same region in which Bloomquist *et al.* (2015) found their largest-effect QTL for oral jaw tooth number in Lake Malawi cichlids.

Several lines of evidence suggest there is shared genetic basis to tooth number divergence on the two jaws of cichlids. The correlation between oral and pharyngeal jaw tooth numbers among individuals in our hybrid cross was highly significant (*r_q_* = 0.35, *P* < 0.0001; Figure 2). Although the size-corrected tooth plate and individual pharyngeal tooth areas did not map to any QTL, the oral and pharyngeal jaws of Lake Victoria cichlids shared a colocalized QTL for tooth number on chromosome 11 (oral tooth number highest LOD = 3.70 and pharyngeal tooth number highest LOD = 3.84; Figure 3). The QTL for oral jaw tooth number in this region was almost significant and pharyngeal jaw tooth number was significant based on permutations that gave a LOD threshold of 3.75 for *P* = 0.05 (Table 1). However, accounting for sex reduced the significance of the pharyngeal tooth number QTL while increasing the association of oral jaw tooth numbers with this region. The QTL for both tooth numbers generally spanned the same region with or without accounting for fish sex (Table 1). Therefore, there could be some interaction between sex and these QTL, but the results are largely robust to the inclusion of sex. Without accounting for sex, the 1.5 LOD interval for oral jaw tooth number ranged from the edge of chromosome 11 to a marker on chromosome 11 at 20 cM. Similarly, the 1.5 LOD interval for pharyngeal jaw tooth number without accounting for sex ranged from a marker located at 3.3 cM on chromosome 11 to the same marker as above at 20 cM. When accounting for sex, markers in this region remained suggestively associated with pharyngeal tooth number but were more clearly associated with oral tooth numbers.

**Table 1:** Tooth number QTL

No Sex				Sex			
Marker	Position	LOD	PVE	Marker	Position	LOD	PVE
Oral tooth number							
10805	3.31	2.10		38662	0.00	2.42	
2874	10.68	3.70	7.30%	2874	10.68	4.08	7.90%
42171	20.63	1.93		42171	20.63	2.34	
Pharyngeal tooth number							
38662	0.00	3.61		2874	10.68	1.37	
56861	15.60	3.84	7.50%	56864	15.69	2.92	5.80%
42171	20.63	2.23		42065	22.68	1.37	

The marker number and the genetic position of these markers in the linkage map for both oral and pharyngeal jaw tooth number QTL are given below when sex is excluded (No Sex) and included (Sex) in analyses. All marker sequences and their inferred positions for the entire linkage map are provided in Table S1 in File S1. The logarithm of odds (LOD) scores for the marker most associated with tooth numbers as well as the markers associated with the 1.5 LOD confidence interval around these peaks are provided. The proportional variance explained (PVE) in tooth number for the QTL are given to the right of the peak LOD score. Based on 1000 permutations, an LOD of 3.75 is considered significant at *P* = 0.05. Therefore, oral tooth number showed a nearly significant QTL without accounting for sex and a clearly significant QTL when accounting for sex. Only when not accounting for sex did pharyngeal tooth number have a significant QTL.

**Figure 4:**
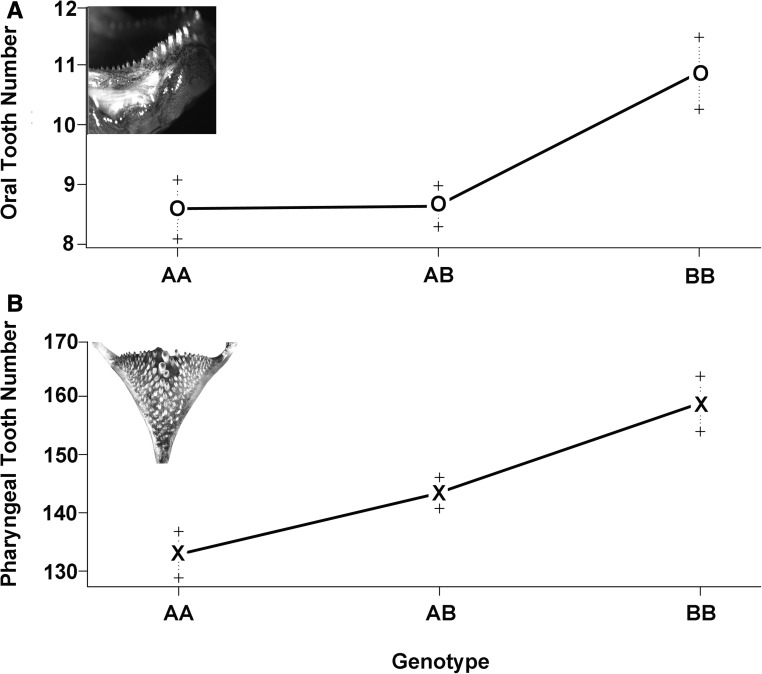
The effect plots of the marker showing the highest LOD score on chromosome 11 for oral jaw tooth number. The B alleles are from *H. nyererei*. The effects in the cross of alleles at this locus are in the same direction for both the oral (A) and pharyngeal jaw (B) tooth numbers.

The one QTL region detected explained up to 8% of the variation in tooth number (oral jaw without sex = 7.3% and oral jaw with sex = 7.9%; pharyngeal jaw without sex = 7.5% and pharyngeal jaw with sex = 5.8%; Table 1). Both of these QTL also influenced numbers in the same direction with the *H. nyererei* alleles contributing to an increase in teeth on both jaws (Figure 4). For example, the homozygotes at the marker showing the highest LOD score on chromosome 11 for oral jaw tooth number for *H. chilotes* had 8.6 ± 0.5 SE teeth, while the *H. nyererei* homozygotes had 10.8 ± 0.5 SE teeth. At this same locus, *H. chilotes* homozygotes had 133 ± 4.1 SE teeth, while *H. nyererei* had 158 ± 5.2 SE teeth. However, the region this QTL bracketed spanned ∼15,340,000 bp in the Tilapia genome.

The shared genetic architecture of tooth number divergence likely has evolutionary consequences for haplochromine cichlid tooth divergence. We could not reject the hypothesis that the correlation coefficient between oral and pharyngeal jaw tooth number in our Lake Victoria hybrid mapping cross, *r_q_* = 0.35, and the phylogenetically corrected correlation among the 36 Malawi cichlids (Fraser *et al.* 2009), *r_p_* = 0.39, were the same.

## Discussion

Tooth numbers do not diverge independently in haplochromine cichlids. QTL for oral jaw tooth number are found in the same region of the genome in the Lake Malawi and the Lake Victoria radiations. In Malawi cichlids, a QTL on chromosome 11 was found to explain 15.5% of the variance in oral jaw tooth number, and this QTL described the most variance of any QTL detected (Bloomquist *et al.* 2015). The single significant QTL region recovered in this study indicates that Lake Victoria cichlids’ oral jaw tooth number divergence is also associated with the same region on chromosome 11. These associations could reflect mutations at similar loci or even shared ancestral polymorphisms (Loh *et al.* 2008; Elmer and Meyer 2011). However, caution is warranted because the credible region for the QTL spans a substantial portion of the chromosome, ∼15 million base pairs (Conte *et al.* 2017). This QTL also brackets at least 500 protein-coding loci that include several genes such as wnt4, fzd6, tuft1a, and crabp2a that could independently influence vertebrate tooth development (Bloomquist *et al.* 2015; Hlusko 2016; Hulsey *et al.* 2016). As genomic resources for these fishes continue to accumulate (Brawand *et al.* 2014; Henning and Meyer 2014; Conte *et al.* 2017), future studies should allow us to more extensively map these genomic associations with tooth number. This should facilitate the rigorous identification of the causal genes and possibly even the specific mutations responsible for tooth number divergence in both radiations of East African cichlids, as well as on the two different jaws of cichlids.

The genomic architectures of cichlid oral and pharyngeal jaw tooth numbers are also not independent. In contrast to the lack of QTL and correlations among several other pharyngeal jaw traits, tooth numbers on both jaws were found to be significantly and positively correlated in our hybrid cross (Figure 2). Because tooth numbers are likely to be highly polygenic traits (Fraser *et al.* 2009; Cleves *et al.* 2014; Hulsey *et al.* 2016), there are undoubtedly a number of other genomic regions that were not detected here that also influence tooth number. Sex also has some interaction with tooth numbers (Table 1). However, it was surprising to find that the one QTL that was recovered separately in the oral and pharyngeal jaws was colocalized and was associated with a similar direction of QTL tooth number effects on both jaws (Figure 3 and Figure 4). This observed integration could provide a putative genomic explanation for why high or low tooth numbers on both jaws are often repeatedly associated with particular feeding ecologies in East African cichlids. In trophically convergent lineages, such as the many piscivores that often have only few teeth, and many herbivorous lineages that generally have many teeth on both jaws (Fryer and Iles 1972; Fraser *et al.* 2009), similar tooth numbers on the two jaws could frequently arise as a byproduct of shared genomic architectures.

How tooth numbers change across species and within our hybrid cross also supports the hypothesis that tooth numbers coevolve owing to shared genomic architectures (Schluter 1996; Brakefield 2006; Hohenlohe and Arnold 2008; Smith 2016). The positive direction and extent to which oral tooth number evolution explained pharyngeal tooth number evolution across a phylogenetic diversity of Malawi cichlids (Fraser *et al.* 2009) was mirrored in the direction and variation with which oral tooth numbers explained pharyngeal tooth numbers in our F2 hybrid cross (Figure 2). Interestingly, when compared to other fishes, this level of integration might be fairly unique. For instance, cypriniform fishes such as *Danio rerio*, the genetic model zebrafish, have completely lost their oral jaw dentition while retaining teeth on only their lower pharyngeal jaw (Huysseune *et al.* 1998; Stock 2001). Teeth on the two jaws of some distantly related cichlids also appear to be able to diverge independently within populations. As an example, the polymorphic Central American cichlid species *Herichthys minckleyi* shows no apparent variation in its oral jaw teeth, but this species is highly polymorphic in pharyngeal jaw tooth numbers (Hulsey *et al.* 2006, 2015). Further comparative studies should allow us to establish whether the hyper-diverse haplochromine cichlids in East Africa generally show greater tooth number integration on their two jaws than do other fishes.

The exceptional rates of diversification and the extent to which haplochromine cichlids have adaptively diverged continue to puzzle evolutionary biologists and provide a challenge to simple explanations for their unparalleled diversity (Fryer and Iles 1972; Liem 1973; Albertson *et al.* 2003; Hulsey *et al.* 2006; Losos 2010; Parnell *et al.* 2012; Wagner *et al.* 2012; Brawand *et al.* 2014; Henning and Meyer 2014). Some East African cichlid species have as few as 40 teeth, while other species have evolved to have over 1000 teeth on each jaw in only a few million years (Fryer and Iles 1972; Friedman *et al.* 2013). Therefore, these fish have radiated to have an order of magnitude more teeth in several orders of magnitude less time than the ∼10,000 extant mammals (Fraser *et al.* 2009). The rapid and correlated evolution among tooth numbers generated from highly linked or even shared genetic loci might be a common way that natural selection and genomics interact to produce the type of exceptional diversity present in the several radiations of East African cichlids (Yoshizawa *et al.* 2013; Miller *et al.* 2014). More generally, serially homologous phenotypes such as the teeth on the two jaws of cichlids that individually face constant selection for trophic specialization, but that share similarities in genomic architectures, could often be the traits that most rapidly diverge during adaptive radiation.

## Supplementary Material

Supplemental material is available online at www.g3journal.org/lookup/suppl/doi:10.1534/g3.115.023366/-/DC1.
